# Resident Parasitoids and Biotic Resistance to Invasive Species in Hawaiʻi, and Implications for Biological Control Programs

**DOI:** 10.1007/s13744-026-01418-2

**Published:** 2026-07-21

**Authors:** Mark G. Wright, Michelle G. Au

**Affiliations:** Dept of Plant and Environmental Protection Sciences, Univ of Hawaiʻi at Mānoa, Honolulu, Hawaiʻi USA

**Keywords:** Island ecosystems, pest management, resident parasitoids, Hymenoptera

## Abstract

Hawaiʻi is subject to disproportionately high rates of biological invasion, including adventive insects. These alien species have been the targets of numerous biological control efforts. The contribution of native species preventing adventives from becoming invasive has received little attention. We analyzed numbers of invasive species utilized by native parasitoids, showing that resident natural enemies infrequently attack alien species, but in some cases, exert significant population control. Purposefully introduced biological control agents likely also contribute to mitigating new invasions. Certain taxa, such as Coleoptera, should be considered priority targets for importation biological control, as they appear highly unlikely to accumulate resident natural enemies.

## Introduction

Island ecosystems are typically more susceptible to the impacts of invasive species than continental regions. In recent decades, rates of adventive alien species accrual in island systems have increased dramatically due to a rise in transportation of goods and tourism. The Hawaiian Islands suffer a disproportionate rate of invasion by insects and other species, with approximately 17–20 new species established annually (Beardsley 1979; Messing & Wright 2006). A number of these species become established pests of agriculture or native ecosystems. Not all accidentally introduced species that establish achieve pest status or become “invasive” by definition of harmfulness. There are several hypotheses addressing establishment and invasiveness, including the “Tens Rule” (Williamson & Brown 1986), which states that one in ten introduced species become established, and one in ten established will become invasive. Recently, Jeschke and Pysek (2018) analyzed the veracity of the Tens Rule, and found that it lacked empirical support. They proposed the 25% invasion rule, specifically for plants and invertebrates. Numerous hypotheses have been proposed to explain why some alien species do not become established and invasive. Enders and Jeschke (2018) list 35 possible mechanisms for biotic resistance, or lack thereof, in new environments. They included four hypotheses related to the roles natural enemies may have in providing resistance to new invasions or why they have limited impact. These hypotheses address reduced impacts of introduced natural enemies on non-native species (Colautti et al. 2004; Eppinga et al. 2006) or enemy release: the absence of natural enemies in the exotic range (Keane & Crawley 2002). Resident natural enemies may provide biotic resistance to exotic species, particularly insects, and thus reduce the likelihood of invasion. These natural enemies can include adventive species that may be biological control agents of pests already present in the environment or native species that form new associations with these exotic introductions. It is possible that “new associations” (Hokkanen & Pimental 1984) between native parasitoids and alien species provide effective biotic resistance in some cases, possibly at different stages of invasion where resident species may have early impacts, while coevolved natural enemies may ensure long-term population regulation. Cornell and Hawkins (1993) examined 87 cases of alien species accruing parasitoid attacks and showed a “vulnerability to parasitism” pattern: herbivorous insects attacked by the most parasitoids in their home range were parasitized by the largest numbers of native parasitoids in invaded regions. It may thus be expected that species with less natural enemies in their native range could have higher invasivity, owing generally to their low susceptibility to natural enemies.

Horrocks et al. (2020) analyzed biotic resistance in New Zealand for introduced Ichneumonidae. They used data on the global known host ranges of introduced Ichneumonidae to predict biotic resistance to potential new pests in New Zealand. Their data showed that 65 exotic Ichneumonidae species were associated with 107 hosts, of which 54 species were pests. They estimated that the Ichneumonidae alone had the potential to suppress 442 pest species that could potentially arrive in New Zealand, thus providing substantial biotic resistance (Horrocks et al. 2020). Wang et al. (2012) quantified mortality of the newly invasive light brown apple moth, *Epiphyas postvittana* (Tortricidae), in California, finding that 12 species of indigenous Diptera and Hymenoptera inflicted high levels of egg, larval, and pupal mortality in moth populations. These examples thus suggest that native and introduced resident natural enemies, specifically parasitoids, may play a substantial role in preventing or reducing invasive insect impacts.

Hawaiʻi has an extensive list of introduced biological control agents (Funasaki et al. 1988; Matsunaga et al. 2019), but also has a diverse, albeit disharmonious, native Hymenoptera fauna that has not been extensively considered a potential source for biotic resistance. In this paper, we analyze the diversity of Hawaiian native species of parasitic wasps, and the numbers of exotic known hosts. We also examine the accrual of resident parasitoids on several economically and ecologically important invasive species in Hawaiʻi and suggest strategies for prioritizing classical biological control program efforts based on these data.

## Materials and methods

We examined the literature for native Hawaiian parasitoid groups within Hymenoptera. Host records for many Hawaiʻi Hymenoptera are poorly known, as most wasp species in taxonomic accounts are obtained through trapping or other forms of adult collection only. In rare cases, efforts have been made to rear parasitoids from known hosts. Published accounts, such as monographs, dealing with parasitoid taxa were examined, and Web of Science searches were conducted using the following search terms: Parasitoid (Hymenoptera) Family; + Hawaii (or Hawaiʻi); and add “host” in separate searches. The literature examined for records of native species of parasitoids exploiting introduced host included Ichneumonidae: Cushman (1944); Bethylidae: Bridwell (1919), Magnacca (2020, 2024), Swezey (1909); Encyrtidae: Beardsley (1976); Eupelmidae: Timberlake (1924); Mymaridae: Triapitsyn and Beardsley (2000), Yang et al. (2002); Sphecidae: Yoshimoto (1959, 1960); and Trichogrammatidae: Oatman et al. (1982). Nishida (2000) was consulted for number of species in each Hymenoptera family, and whether they were native, purposefully, or accidentally introduced.

We also examined recently (since 2005) established invasive species (Table 1), and the resident natural enemies they have accrued, based on published and unpublished data. These species all have substantial negative impacts on Hawaiian agriculture or native ecosystems with no imported natural enemies released for their control in Hawaiʻi. The eight species analyzed are the most significant invasive insect pests in Hawaiʻi during the past 20 years causing economic and ecological damage.

**Table 1:** Recent invasive species in Hawaiʻi, resident natural enemy (parasitoid) accrual, and known natural enemies (parasitoids) in the native provenance. Species are listed based on year of detection (2005–2018)

Insect taxon	Site of damage (year of detection)	Accrued natural enemies in Hawaiʻi	Natural enemies in native provenance
*Quadrastichus eyrthinae*, Eulophidae, erythrina gall wasp^1^	Endemic and introduced landscape *Erythrina* spp. (2005)	None	Specialized parasitoid wasps^1^
*Acanthococcus ironsidei*, Eriococcidae, macadamia felted coccid^2,3^	Macadamia nut trees (2005)	1 parasitoid^2,3^	Specialized parasitoid in Australia^4^
*Hypothenemus hampei*, Curculionidae, coffee berry borer^5,6^	Coffee beans (2010)	None	Multiple specialized parasitoids in Africa^5^
*Paratachardina lobata*, Kerriidae, lobate lac scale^7,8^	Polyphagous; native plants including *Hibiscus* spp. (2012)	None	Encyrtidae, Eulophida^9^
*Oryctes rhinoceros*, Scarabaeidae, coconut rhinoceros beetle^10^	Palms, including endemic species (2013)	None	Parasitoid Diptera and Hymenoptera
*Prosapia bicincta*, Cercopidae, two-lined spittlebug^11^	Pastures with African grasses (2016)	None	Unknown
*Arcte coerula*, Noctuidae, ramie moth^12^	Endemic & non-native nettles (2018)	2 egg parasitoids; 2 larval parasitoids^12^	Unknown
*Pseudacysta perseae,* Tingidae, avocado lace bug^13^	Avocado leaves (2018)	None	Generalist parasitoids and predators

^1^ Ramadan et al. 2023; ^2^ Wright & Conant 2009, ^3^ Pulakkatu-thodi et al. 2022, ^4^ Yalemar et al. 2023; ^5^ Yousuf et al. 2021, ^6^ Honsberger 2024; ^7^ Kaufman & Higashi 2015, ^8^ Cheng et al. 2018, ^9^ Pemberton 2003; ^10^ Russo 2024; ^11^ Wilson et al. 2023; ^12^ Au & Wright 2022; ^13^ Bosch et al. 2024

## Results

**Fig. 1:**
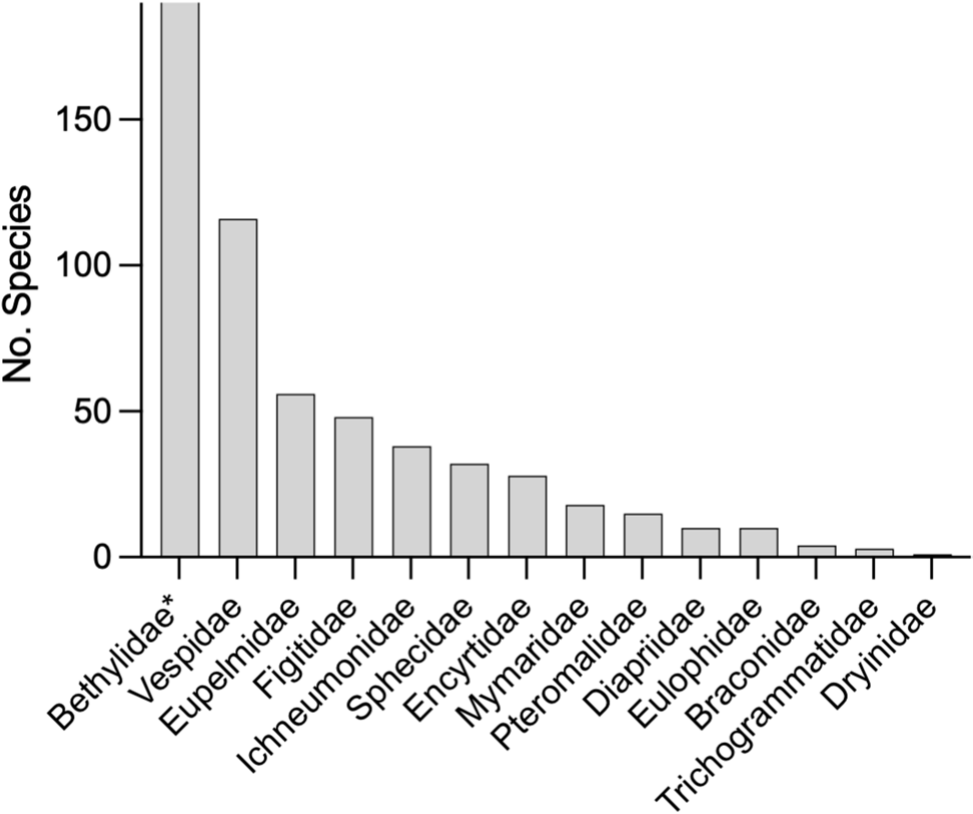
Number of native species of parasitoid and predatory Hymenoptera recorded in Hawaiʻi. *There are more than 500 species of Bethylidae in Hawaiʻi, truncated here

**Fig. 2:**
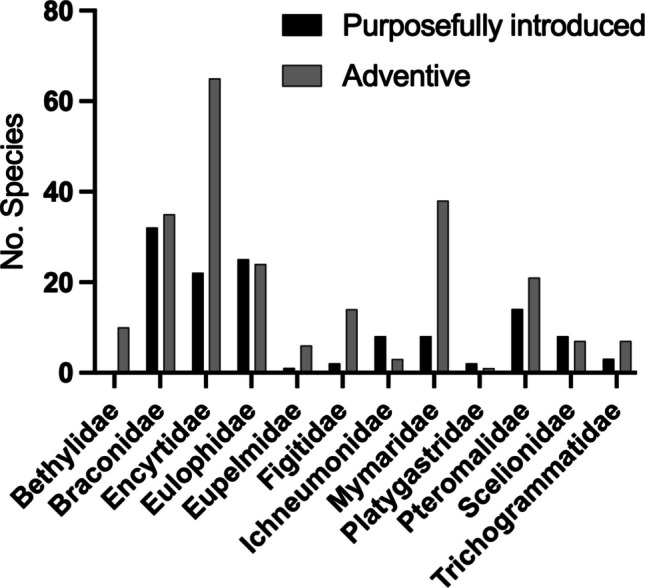
Number of adventive (accidently introduced) and purposefully introduced Hymenoptera in Hawaiʻi. Data from Nishida (2000) and Matsunaga et al. (2019)

Numerous species of Hymenoptera have been described from Hawaiʻi (Fig. 1). Bethylidae is the most speciose family with > 500 described species, at least five times more than the next most species-rich parasitoid family, Eupelmidae. However, most families are quite depauperate in native species (Fig. 1). Numbers of purposefully and accidentally introduced Hymenoptera are shown in Fig. 2.

**Fig. 3:**
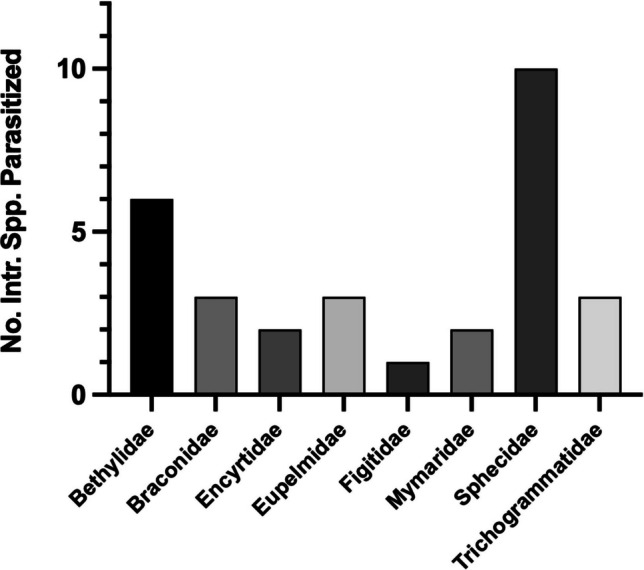
Number of species of introduced (alien) hosts recorded for native Hawaiian Hymenoptera by family

Although Vespidae are not parasitoids that would be considered in biological control programs, they likely provide biotic resistance as predators and deserve greater attention to determine their prey range and to what extent it includes alien species. Other predatory Hymenoptera in Hawaiʻi such as Sphecidae have been recorded preying upon and parasitizing several alien species (Fig. 3, Yoshimoto 1958, 1960).

There are few records of alien species as hosts for native parasitoids or predators (Fig. 3). These records are likely a poor reflection of the true extent of parasitism by native Hymenoptera.

Over the last two decades, Hawaiʻi has been impacted by several new important invasive species (Table 1). Of the eight listed, only two have accrued resident parasitoids. Ramie moth, *Arcte coerula*, populations may be effectively constrained by resident egg parasitoids, Trichogrammatidae and Encyrtidae of unknown origin, and the impacts of generalist predators (Michelle Au, 2025, field surveys [unpublished field data]).

## Discussion

The Hawaiian insect fauna is considered an extreme example of a disharmonic fauna (Howarth 1979), clearly reflected in the distribution of species richness within the parasitic Hymenoptera. It is therefore likely that groups that provide substantial biotic resistance potential in other places, e.g., Ichneumonidae in New Zealand (Horrocks et al. 2020), will be less significant in Hawaiʻi where there are few species, even those that were purposefully and unintentionally introduced (Fig. 2). Few researchers over the decades have explored host associations rather than concentrating on taxonomic accounts using wasps collected as adults in order to gain a better understanding of these relationships. Recent work, e.g., Honsberger (2024), Honsberger et al. (2024), has described numerous species of cryptic parasitoids utilizing Scolytinae beetles. However, it is uncertain whether these new species are native or introduced. The systematics of native Hawaiian Hymenoptera and unknown introduced species deserves greater research attention, likely revealing many new species with potential for providing biotic resistance.

A notable example of endemic Hawaiian parasitoids suppressing populations of an invasive species is of the two-spotted leafhopper, *Sophonia rufofascia* (Cicadellidae) (Johnson et al. 2001; Yang et al. 2002). *Sophonia rufofascia* became widely distributed in native vegetation in Hawaiʻi since its discovery in 1987, causing significant impacts. By 2002, endemic *Polynema* spp. (Mymaridae) were inflicting substantial mortality on their populations (Yang et al. 2002).

Native parasitoids in Hawaiʻi may be largely specialized on native hosts, having evolved in isolation with them, and thus may have less potential for expanding their host ranges to adventive species than generalist would. Purposefully introduced parasitoids in Hawaiʻi have been recorded using alternative alien hosts than the intended target species, with some groups more susceptible to these host range expansions. For example, approximately 45% of alien Hemiptera (scale insects, mealy bugs), 10% of alien Diptera, and 70% of alien Lepidoptera are hosts to introduced parasitoids in Hawaiʻi (Funasaki et al. 1988). Notably, only 2% of alien Coleoptera species are used by introduced parasitoids (Funasaki et al. 1988). Most diverse local species-pools will provide the greatest probability of successful resident biotic resistance. On the continental USA, in Delaware alone, at least 15 species of Braconidae parasitizing Cerambycidae were found in a 7-year study (Golec et al. 2020), providing potential for biological control of invasive Cerambycidae. No native parasitoids of Cerambycidae have been reported from Hawaiʻi.

As noted above, introduced Lepidoptera are disproportionately susceptible to resident parasitoids. While macadamia felted coccid, *Acanthococcus ironsidei*, is parasitized by a resident species, *Encarsia lounsburyi* (Aphelinidae), the impact on pest populations is minimal (Wright & Conant 2009; Gutierrez-Coarite et al. 2018). The coffee berry borer, *Hypothenemus hampei*, has not accrued any resident parasitoids, despite the presence of numerous species of parasitoids of Scolytinae in Hawaiʻi (Honsberger 2024). Both latter species have specialized parasitoids in their native ranges that provide effective population regulation.

Specific traits of alien species likely dictate their susceptibility to native natural enemies, as noted by Cornell and Hawkins (1993). For example, the hard shield on lobate lac scale, *Paratachardina lobata*, may protect the insect effectively from non-specialist parasitoids that are present around plants attacked by this insect; the breeding sites for coconut rhinoceros beetle, *Oryctes rhinoceros*, require larval parasitoids to be adapted in finding hosts in deep layers of decaying plant material. The absence of any evolutionary interactions between native parasitoids and natives closely related to these alien species likely also plays a significant role. For example, no Cercopidae (spittlebugs) are native to Hawaiʻi, and native parasitoids have had no opportunity for preadaptation to breaching their spittle masses, which provide effective defense to nymphs. Host specific parasitoids do not readily undergo adaptative selection to adopt new hosts (Wright & Bennett 2018; Hopper & Leung 2026), so endemic species may be unlikely to adapt to alien species and generalist parasitoids are more likely to provide biotic resistance.

Much of the literature examined for this review dates back to the early 1900 s, surely a contributing factor for the few new host records of endemic parasitoids. Invasive insects may take long periods to become associated with resident natural enemies, e.g., Horrocks et al. (2020) showed a consistent linear rate of accrual of new associations over 108 years. However, numerous alien pest species are continuously investigated in Hawaiʻi, revealing few new records of resident parasitoids. For example, Honsberger et al. (2022) reported a new larval parasitoid of diamondback moth (*Plutella xylostella*), a prominent pest species which has been present in Hawaiʻi since the late 1800 s, and one that receives consistent attention in the state. Despite the parasitoid (*Oomyzus sokolowskii*, Eulophidae) being very small and probably easily overlooked, it is surprising that it had not been previously detected from this host, unless it is a recent adventive species in Hawaiʻi. Although there are still many resident parasitoids that have yet to be discovered in Hawaiʻi with certain groups receiving more attention than others, looking at recent invasives helps to determine some of these potential associations for biotic resistance.

## Conclusion

While Hawaiʻi has a significant native parasitoid fauna, it is a highly disharmonic one, with most groups poorly represented. Parasitoid families that are well represented in other regions are poorly represented in Hawaiʻi. Some of these native parasitoids have expanded their host range to include alien species, and in a few cases, the invasive species have been effectively suppressed. Some groups of alien insects do incur considerable parasitism from previously introduced biological control agents for other pest species. However, many alien species do not accumulate significant resident natural enemy impacts. Those species that escape the impacts of resident natural enemies are likely able to do so because of specific traits and behaviors that make them successful. It is suggested that new invasions of certain groups, for example, Coleoptera, should be considered priorities for classical biological control efforts due to low parasitism by resident natural enemies, although this is based on limited empirical support. A better understanding of the native parasitoid groups in Hawaiʻi and their host range breadth will allow us to better predict the likelihood of biotic resistance and the need for importation of natural enemies.
